# Comparative evaluation of NOTCH signaling molecules in the endometrium of women with various gynecological diseases during the window of implantation

**DOI:** 10.22038/ijbms.2019.32961.7874

**Published:** 2019-04

**Authors:** Fatemehsadat Amjadi, Ensieh Salehi, Zahra Zandieh, Mandana Rashidi, Sara Taleahmad, Mojgan Javedani masrour, Reza Aflatoonian, Mehdi Mehdizadeh

**Affiliations:** 1 Shahid Akbarabadi Clinical Research Development Unit (ShACRDU), Department of Anatomical Sciences, School of Medicine, Iran University of Medical Sciences, Tehran, Iran; 2 Cellular and Molecular Research Center, Faculty of Advanced Technologies, Department of Anatomical Sciences, Iran University of Medical Sciences, Tehran, Iran; 3 Department of Anatomy, School of Medicine, Tehran University of Medical Sciences, Tehran, Iran; 4 Shahid Akbarabadi Clinical Research Development Unit (ShACRDU), IVF Center, Iran University of Medical Sciences, Tehran, Iran; 5 Department of Molecular Systems Biology, Cell Science Research Center, Royan Institute for Stem Cell Biology and Technology, ACECR, Tehran, Iran; 6 Research and Clinical Center of Gynecology and Fertility, Shahid Akbarabadi Hospital, Iran University of Medical Sciences, Tehran, Iran; 7 Department of Endocrinology and Female Infertility at Reproductive Biomedicine Research Center, Royan Institute for Reproductive Biomedicine, ACECR, Tehran, Iran

**Keywords:** Endometriosis, NOTCH signaling, PCOS, Repeated implantation-failure (RIF), Window of implantation

## Abstract

**Objective(s):**

NOTCH signaling pathway is well known for its role in cell fate, cell survival, cell differentiation, and apoptosis. Some of the NOTCH signaling genes are critical for endometrial function and implantation in animals and appear to play a similar role in humans. The purpose of the current study was to investigate the potential roles of some main components of the NOTCH family in human endometrium during implantation period in common gynecological diseases.

**Materials and Methods:**

Endometrial NOTCH receptors NOTCH1, 3, 4 and ligand JAG1, 2 and survivin mRNA expression were investigated using the Q-PCR technique and the amount of the JAG1, 2 proteins was also determined by Western blot. Samples were obtained from 12 patients with endometriosis, 12 patients with repeated implantation failure (RIF), 12 patients with Polycystic Ovary Syndrome (PCOS) and 10 healthy fertile women as a control group. Data were analyzed using SPSS version 18. Group comparisons were performed by one-way ANOVA or Kruskal-Wallis.

**Results:**

All patient groups failed to show the expected mid-luteal increase in NOTCH1, JAG 1, 2, and survivin expression as documented in the control group. Moreover, a significant rise in NOTCH3 expression levels was found only in PCOS women. There was a direct correlation between gene expression and protein level for JAG 1, 2.

**Conclusion:**

Aberrant NOTCH signaling molecules expression suggests that altered development of the endometrium at the molecular level may be associated with the impaired decidualization and implantation failure in gynecological disorders such as endometriosis, PCOS, and RIF.

## Introduction

Implantation is a key part of early pregnancy in which blastocyst adheres and penetrates into the wall of the receptive endometrium. A complex sequence of cellular and molecular changes should take place at the maternal endometrium in order to permit the embryo to invade the decidua (1). Until recently, the exact factors mediating the endometrial receptivity were not fully understood. Among these, NOTCH signaling genes are supposed to be important factors in the endometrial function (2).

NOTCH signaling is an evolutionarily conserved pathway, which regulates cell proliferation, cell fate determination, cell invasion, differentiation, and apoptosis (3, 4). Each of these processes absolutely exhibits an important role in endometrial remodeling. In mammals, NOTCH signaling family includes four NOTCH receptors (NOTCH1-4), five ligands, three Delta-like proteins (DLL1,3,4) and two Jagged proteins (JAG1,2). Both the NOTCH receptors and ligands are single-pass transmembrane proteins, hence, NOTCH signaling is activated upon cell-to-cell contact (5). Receptor/ligand interaction at the cell surface, activates proteolytic cleavage of the receptor, leading to the release of intracellular NOTCH receptor domain, which enters the cell nucleus and induces the NOTCH target genes expression (6, 7), including survivin (8).

There are only a few reports concerning the expression pattern and role of the NOTCH pathway in the human endometrium. In physiological conditions, significant increase in endometrial NOTCH1 and decrease in NOTCH4 during the luteal phase, suggests that NOTCH-4 likely contributes more to the controlling proliferation, while, NOTCH-1 more likely involves in the differentiation programming (2). In addition, an up-regulated expression of JAG1 and DLL4 as the NOTCH ligands localized in the endometrium have been reported during the mid-luteal phase, which implies their involvement in the endometrial receptivity(9).

It has been shown that endometrial remodeling, decidualization and embryo implantation could be modulated by the NOTCH signaling in mice. Afshar *et. al.* indicated a decrease in proliferation and up-regulation of apoptosis-associated genes via inhibition of NOTCH1 signaling during decidualization (10). PCOS (40%), endometriosis (50%), repeated implantation failure (10%) are three common causes of female infertility (11-13).

Women with PCOS, endometriosis, and RIF have been reported to exhibit an altered expression pattern of receptivity markers during the period of implantation (14-17). Therefore, the aim of this study was to evaluate the expression of the NOTCH receptors (NOTCH1, NOTCH2, NOTCH3), ligands (JAG1and JAG2) and target gene, and survivin, in the endometrium of patients with PCOS, endometriosis, repeated implantation failure (RIF) and healthy fertile women during the window of implantation.

## Materials and Methods

***Patients and tissue selection***

This prospective study was performed, during a 12 months’ period from September 2015 to October 2016. Signed informed consent was taken from each participant and the study was approved by the local Ethics Committee at Iran University of Medical Sciences (IUMS, no 19950). Human endometrial tissue samples were obtained from PCOS women (n=12), endometriosis (n=12), RIF patients (n=12), and healthy fertile women in the secretory phase (on days 3–5 after ovulation, n=10). Patients with chronic oligo/anovulation (cycle length >35 days and < 6 months), hyperandrogenism, and polycystic ovaries were identified as PCOS (consistent with Rotterdam criteria). The timing of the LH surge, ultrasound scanning, and histological evaluation, confirmed that samples were harvested during the luteal phase. The mean age of the subjects was 26 years (range: 18–35). Women with any pathological uterine disorder and endometrial hyperplasia were not included. They also had not used hormonal medications or an intrauterine contraceptive device in the previous three months. Under sterile conditions, endometrial biopsies were collected using Pipelle catheters (Pipelle, Laboratoire CCD, France). Each endometrial sample was cut into two portions: for RNA and protein extraction, the first part was put inside a Cryo tube containing 1 ml RNA later (Ambion, Austin, TX, USA). Another part was fixed in formalin to perform histological dating.

**Figure 1:**
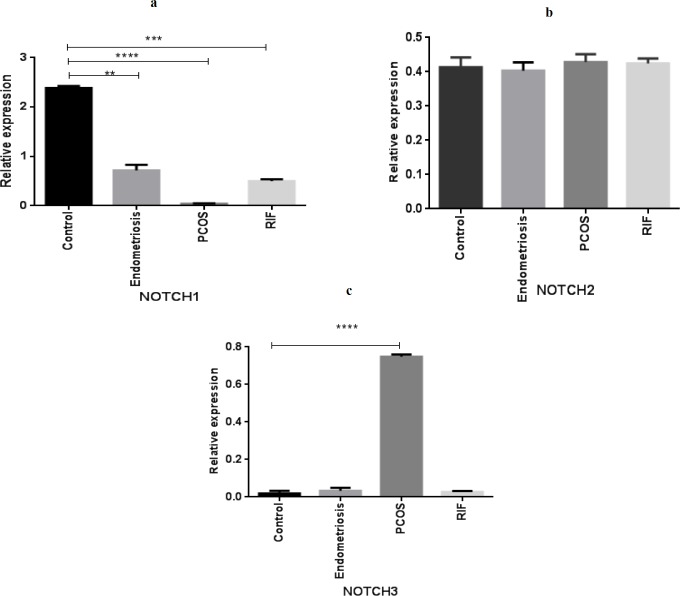
Gene expression pattern of NOTCH receptors (NOTCH1, NOTCH2, NOTCH3) in women with polycystic ovary syndrome(PCOS), endometriosis, and repeated implantation failure(RIF) groups compared with the control group (mean± SEM)

** represents significance at *P<*0.01, *** represents significance at *P<*0.001

****represents significance at *P<*0.0001

**Figure 2:**
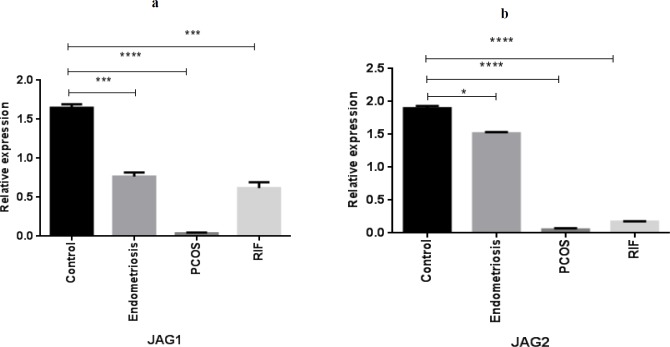
Gene expression pattern of NOTCH ligands (JAG1, JAG2) in women with polycystic ovary syndrome (PCOS), endometriosis, and repeated implantation failure (RIF) groups compared with the control group (mean± SEM)

* represents significance at *P<*0.05, *** represents significance at *P<*0.001

****represents significance at *P<*0.0001

**Figure 3:**
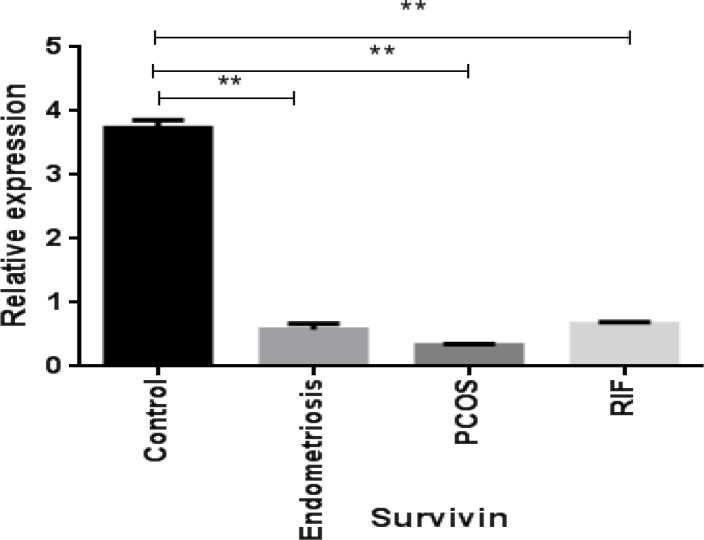
Gene expression pattern of NOTCH target gene survivin in women with polycystic ovary syndrome (PCOS), endometriosis and repeated implantation failure (RIF) groups compared with the control group (mean±SEM)

** represents significance at *P<*0.01

**Figure 4:**
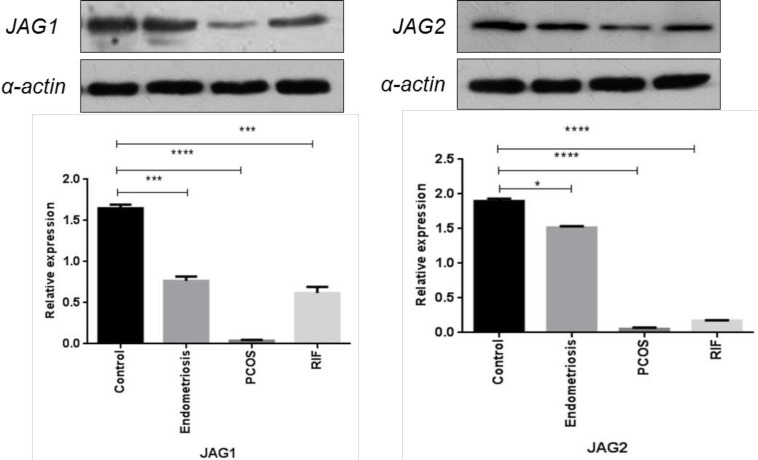
Protein expression pattern of NOTCH ligands (JAG1, JAG2) in women with polycystic ovary syndrome (PCOS), endometriosis, and repeated implantation failure(RIF) groups compared with the control group (mean±SEM)

* represents significance at *P<*0.05, *** represents significance at *P<*0.001

****represents significance at *P<*0.0001

**Table 1:** The primer sequences (5'-3') used in quantitative real-time polymerase chain reaction (qRT-PCR)

Gene symbol	Forward primer	Reversed primer
β-actin	TGACCCAGATCATGTTTGAGACC	GGAGGAGCAATGATCTTGATCTTC
NOTCH1	AGAAGATGCTCCAGCAACACA	GCAAGTCTCCTACAAAACACGG
NOTCH2	AATGAGTGTCTGAGTGAACCCT	GACTCCATCAAATCCTGCCTG
NOTCH3	CTCATGGTATCTGCACCAACCT	GGGTCACAGTCATTGATGTCCT
JAG1	CTGTGGCTTGGATCTGTTGCT	CGTTGTTGGTGGTGTTGTCCT
JAG2	CCTCTGCCTTGCTACAATGGTG	GCGATACCCGTTGATCTCATCC
Survivin	GATTTGATTCGCCCTCCTCCC	AACAGCCGAGATGACCTCCA

***RNA extraction, cDNA synthesis, and reverse transcription polymerase chain reaction (RT-PCR)***

Total RNA was isolated from endometrial tissue samples by homogenization in Trizol reagent (Sigma Pool, UK), following the manufacturer’s instructions. To remove genomic DNA contamination, RNA samples were purified with DNase I (Fermentas, Sankt Leon-Rot, Germany). RNA was reverse transcribed into cDNA using SuperScript First-Strand Synthesis System (200 U/μl, fermentase). Negative controls were prepared as non-reverse transcribed controls (RT controls). The RT-PCR was done using prepared cDNA, Platinum Blue PCR SuperMix (Invitrogen, Paisley, Scotland, UK), and the forward and reverse primers for NOTCH1-3, JAG1, JAG2 and survivin (Metabion, Martinsried, Bavaria, Germany). The used primer sequences are described in Table 1. The RT-PCR conditions for 40 cycles were as follows: 95 ^°^C for 30 sec, 60 ^°^C for 1 min, and 72 ^°^C for 1 min. To separate PCR products, agarose gel (1.2%) electrophoresis and visualizing via an ultraviolet transillumination were carried out. The identity of the amplified product was confirmed by the sequencing of the RT-PCR products. β-actin was considered as a reference gene (18).

***Quantitative real-time polymerase chain reaction (qPCR)***

All transcripts were quantified in triplicates using an ABI Prism 7300 apparatus (Applied Biosystems, Foster City, California, USA). The total reaction volume was 20 μl containing 250 ng cDNA, 5 pmol of each primer, and SYBR Green reagent (Applied Biosystems) with ROX dye as a passive control for signal intensity. Amplification was carried out at 95 ^°^C for 30 sec, 60 ^°^C for 30 sec, and 72 ^°^C for 30 sec. Melting curves of PCR reactions were used to check the formation of nonspecific products and primer-dimer. The efficiency of the primers was tested by the standard curves.

To analysis qPCR results, the comparative CT method was utilized. ΔCT was defined as the difference between the number of cycles required for amplification of the candidate gene and the human β-actin (reference gene selected). Then, ΔΔCT was calculated as the difference between the groups and 2^-ΔΔCT^ was reported as mRNA fold change (FC) (18).

***Western blotting***

A 50 µg protein aliquot from each sample was separated on SDS-polyacrylamide gel and then blotted onto polyvinylidene fluoride (PVDF) membranes (Bio-Rad, USA). The membranes were blocked with 5% BSA and 0.1% Tween-20 (Sigma-Aldrich, St. Louis, MO, USA). Then, the membranes were incubated with the primary anti-JAG1 antibody (1:100; Abcam ab7771, Cambridge, UK), anti-JAG2 antibody (1:100; Abcam ab109627, Cambridge, UK) and β-actin (1:200; Sigma-Aldrich, St. Louis, MO, USA) overnight at 4 ^°^C. The blots were then washed with TBST, incubated with horseradish peroxidase (HRP)-conjugated secondary antibody (1:200; Abcam, ab7086) for 1 hr at room temperature. After washing with TBST, immunodetection was achieved with the chemiluminescent peroxidase substrate (ECL Advance, GE Healthcare, UK) and blots were exposed to x-ray films (GE, 28906835). Consequently, the films were scanned with a densitometer (GS-800, Bio-Rad, USA). The signal intensity of JAG1 and JAG2 relative to the density of β-actin band was computed using the Image G software. As a negative control, the blots were treated with non-immunized serum as the primary antibody.

***Statistical analysis***

Data were analyzed using SPSS version 18 and are described as mean ± SD. Distribution of data was checked with the Kolmogorov-Smirnov test. Between groups comparisons were performed by one-way ANOVA for the data with normal distribution and by the Kruskal-Wallis for non-parametric data. Statistical significance was set at *P<*0.05.

## Results

***Expression of NOTCH receptors in the endometrium of women with gynecological diseases compared with the healthy fertile women***

qPCR analysis indicated a significant decrease in NOTCH1 mRNA expression levels of women with PCOS, endometriosis and RIF groups compared with the healthy group during the implantation window (Figure 1). As shown in Figure 2, mRNA expression of NOTCH2 has no statistically significant difference between the women with different gynecological diseases and the control group (Figure 2). In addition, NOTCH3 was significantly up-regulated only in the PCOS group compared with the healthy fertile women (Figure 3).

***Expression of NOTCH ligands in the endometrium of women with gynecological diseases compared with the healthy fertile women***

A significantly lower expression of JAG1 was observed in PCOS (*P*<0.0001), endometriosis (*P*<0.001), and RIF (*P*<0.001) groups compared with the healthy fertile women (Figure 2, a). Furthermore, JAG2 gene expression was significantly down-regulated in the endometrium of PCOS (*P*<0.0001), endometriosis (*P*<0.05), and RIF (*P*<0.0001) groups compared with control subjects (Figure 2, b). 

***Expression of the NOTCH target gene, survivin, in the endometrium of women with gynecological diseases compared with the healthy fertile women***

As shown in Figure 3, survivin gene expression was decreased in the endometrial samples of PCOS women, and endometriosis and RIF groups compared with healthy women.

***Immunoblotting analysis of NOTCH ligands in the endometrium of women with gynecological diseases compared with the healthy fertile women***

Western blot analysis showed a significantly lower JAG1 and JAG2 protein expression in the endometrial samples of PCOS patients, and endometriosis and RIF groups compared with the healthy fertile ones (Figure 4). 

## Discussion

In the current study, the expression pattern of NOTCH signaling molecules as potential biomarkers was assessed in the endometrial samples taken during the mid-luteal phase of women with endometriosis, PCOS, and RIF. Our results showed that NOTCH pathway molecules including NOTCH1, 3, JAG1, 2, and survivin are differently expressed in the patient groups compared with healthy controls. A large body of evidence suggests that aberrant endometrial receptivity may attribute to the undesired reproductive outcomes in common gynecological disorders including PCOS, endometriosis, and RIF (19-22). Various biological molecules have been proposed to contribute to the impairment of decidualization and endometrial receptivity (23). 

NOTCH pathway mediates cell differentiation, cell progression, and cell death and regulates angiogenesis (24-26). All of these processes are critical for successful implantation (1, 27). Su *et. al.* indicated that endometrial NOTCH molecules attenuate in patients with endometriosis. They suggested that the decreased NOTCH signaling contributes to the decidualization defects and therefore insufficient uterine receptivity (28).

Inconsistently, the current study demonstrated that NOTCH1, JAG-1, JAG-2, and survivin, as a down target molecule of NOTCH pathway, significantly decrease in women with PCOS, endometriosis, and RIF. It is documented that NOTCH1 is involved in decidual angiogenesis and endometrial differentiation, while NOTCH3 and 4 control the proliferation (2, 29). Animal studies have shown that NOTCH1 silencing leads to the decrease in IL-11 and IGFBP1 as two main human decidualization biomarkers (10). Moreover, NOTCH1 deficiency results in the reduction of bone morphogenetic protein2 (bmp2) and wnt4 in the mouse uterus, which are necessary for the decidualization (10). A recent study has revealed that NOTCH1 and NOTCH ligands including JAG1 and DLL1 are down-regulated in the endometrium of women with unexplained infertility during the implantation window compared with the fertile subjects (30). DLL1 changes integrin α6 and integrin β1 expression, which have essential roles in the embryo implantation (31). The expression of JAG1 was increased from proliferative into secretory phase, which proposes a responsibility for JAG1 in the endometrial receptivity (2, 30), and the endometrial expression of JAG2 was decreased in endometriosis, which is contributed to the decidualization failure (28).

Interestingly, we found NOTCH-3 increased in PCOS exclusively. Previous studies revealed that the NOTCH system plays a pivotal role in maintaining balance between cell proliferation and cell death (32). It has been shown that NOTCH3 increases endometrial cell proliferation, and NOTCH1 promotes differentiation (2, 29). It seems both underexpression of NOTCH1, as well as overexpression of NOTCH3, may have detrimental effects on uterine receptivity in PCOS patients. Also, increased NOTCH3 expression may be related to the elevated endometrial thickness and hyperplasia that is seen in women with PCOS.

## Conclusion

In summary, our findings illustrate that dysregulated NOTCH signaling molecules during the window of implantation may be associated with implantation problems and consequently poor outcomes observed in endometriosis, PCOS, and RIF. However, our knowledge is still limited due to the complexity of this pathway. Hence, much remains to be discovered about the role of the NOTCH signaling pathway and its changes during the mid-luteal phase in normal and disease conditions.
